# Widespread Changes in White Matter Microstructure after a Day of Waking and Sleep Deprivation

**DOI:** 10.1371/journal.pone.0127351

**Published:** 2015-05-28

**Authors:** Torbjørn Elvsåshagen, Linn B. Norbom, Per Ø. Pedersen, Sophia H. Quraishi, Atle Bjørnerud, Ulrik F. Malt, Inge R. Groote, Lars T. Westlye

**Affiliations:** 1 Department of Psychosomatic Medicine, Institution of Oslo University Hospital, Oslo, Norway; 2 Department of Neurology, Institution of Oslo University Hospital, Oslo, Norway; 3 The Intervention Centre, Institution of Oslo University Hospital, Oslo, Norway; 4 Norwegian Centre for Mental Disorders Research (NORMENT)/KG Jebsen Centre for Psychosis Research, Institution of Oslo University Hospital, Oslo, Norway; 5 Department of Research and Education, Institution of Oslo University Hospital, Oslo, Norway; 6 Institute of Clinical Medicine, University of Oslo, Oslo, Norway; 7 Department of Psychology, University of Oslo, Oslo, Norway; 8 Department of Physics (AB), University of Oslo, Oslo, Norway; 9 Barnard College, Columbia University, New York, NY, United States of America; University of Pécs Medical School, HUNGARY

## Abstract

**Background:**

Elucidating the neurobiological effects of sleep and waking remains an important goal of the neurosciences. Recently, animal studies indicated that sleep is important for cell membrane and myelin maintenance in the brain and that these structures are particularly susceptible to insufficient sleep. Here, we tested the hypothesis that a day of waking and sleep deprivation would be associated with changes in diffusion tensor imaging (DTI) indices of white matter microstructure sensitive to axonal membrane and myelin alterations.

**Methods:**

Twenty-one healthy adult males underwent DTI in the morning [7:30AM; time point (TP)1], after 14 hours of waking (TP2), and then after another 9 hours of waking (TP3). Whole brain voxel-wise analysis was performed with tract based spatial statistics.

**Results:**

A day of waking was associated with widespread increases in white matter fractional anisotropy, which were mainly driven by radial diffusivity reductions, and sleep deprivation was associated with widespread fractional anisotropy decreases, which were mainly explained by reductions in axial diffusivity. In addition, larger decreases in axial diffusivity after sleep deprivation were associated with greater sleepiness. All DTI changes remained significant after adjusting for hydration measures.

**Conclusions:**

This is the first DTI study of sleep deprivation in humans. Although previous studies have observed localized changes in DTI indices of cerebral microstructure over the course of a few hours, further studies are needed to confirm widespread DTI changes within hours of waking and to clarify whether such changes in white matter microstructure serve as neurobiological substrates of sleepiness.

## Introduction

Sleep is an enigmatic, evolutionarily conserved process required for human health and functioning [1–3]. Lack of sleep causes substantial impairments across cognitive domains in healthy subjects [4, 5] and disturbances in the sleep-wake cycle are frequently observed in individuals with neuropsychiatric disorders [6, 7]. In addition, sleep deprivation can have rapid antidepressive effects in mood disorders [8, 9]. Therefore, elucidating the neurobiological effects of sleep and waking remains an important goal of the basic and clinical neurosciences.

A longstanding and widely held belief is that sleep is restorative [10, 11]. In support of this hypothesis, increased brain expression of genes regulating macromolecule biosynthesis has consistently been found in flies, rodents, and birds during sleep [12–15]. In particular, accumulating evidence indicates that sleep is associated with elevated transcription of genes involved in synthesis and maintenance of cell membrane lipids and myelin in the brain [14, 16, 17]. Consistent with a role for sleep in membrane lipid homeostasis, sleep deprivation caused a marked increase in breakdown of membrane phospholipids of neurons *in vitro* and *in vivo* [18]. Together, these findings indicate that sleep is important for cell membrane and myelin maintenance in the brain and that these structures might be particularly susceptible to insufficient sleep [17, 18].

Diffusion tensor imaging (DTI) is a magnetic resonance imaging (MRI) technique that is sensitive to water diffusion in biological tissues and to axonal membrane and myelin alterations in the brain [19, 20]. Because water diffusion is higher parallel than perpendicular to white matter (WM) axons, causing directional or anisotropic diffusion, DTI enables indirect investigation of WM microstructure [20, 21]. The indices of WM microstructure obtained from DTI include fractional anisotropy (FA), which reflects the degree of anisotropic diffusion, and axial diffusivity (AD) and radial diffusivity (RD), i.e., measures of diffusion along and across WM tracts, respectively [19]. Despite widespread and increasing use of DTI in the neurosciences, it is unknown whether DTI indices of cerebral WM microstructure show sensitivity to lack of sleep.

Although there is a scarcity of human studies assessing the effects of sleep deprivation on WM microstructure, a recent study examined whether a day of waking was associated with changes in brain DTI parameters of healthy volunteers [22]. Here, Jiang *et al*. found widespread decreases in WM RD, AD, and MD from morning to evening, thus indicating that brain DTI changes can take place within hours of waking. Changes in WM functioning after a day of waking and sleep deprivation have also been suggested by recent functional connectivity MRI studies [23–25]. Shannon *et al*. observed diurnal changes in connectivity between medial temporal lobe regions and the cortex [23], whereas De Havas *et al*. [24] and Sämann *et al*. [25] found that default-mode network integrity was reduced after sleep deprivation.

In the present study of healthy humans, we tested the hypotheses that a day of waking followed by a night of sleep deprivation would be associated with changes in cerebral WM DTI parameters, possibly reflecting axonal membrane and myelin alterations, and that larger DTI changes after sleep deprivation would be associated with greater sleepiness.

## Methods and Materials

### Ethics Statement

This study was approved by the Regional Ethical Committee of South-Eastern Norway (REK Sør-Øst) and was conducted according to the principles expressed in the Declaration of Helsinki. All subjects provided written informed consent to participate.

### Participants

Twenty-one healthy adult males (mean [SD] age, 22.1 [2.1] years) were recruited through local advertising. Sixteen participants were right-handed (76.2%) and 18 (85.7%) were university students. Exclusion criteria were: history of sleep disorder, neurological or other chronic somatic disorder, psychiatric illness, alcohol or drug use disorder, previous head injury with loss of consciousness for more than one minute, and metallic implants. All subjects had a regular sleep-wake cycle and reported an average of 7.3 ± 1.2 hours of sleep per night the week before the study and 6.3 ± 1.1 hours of sleep the night before participating in the study.

### Study Protocol

**Fig 1:**
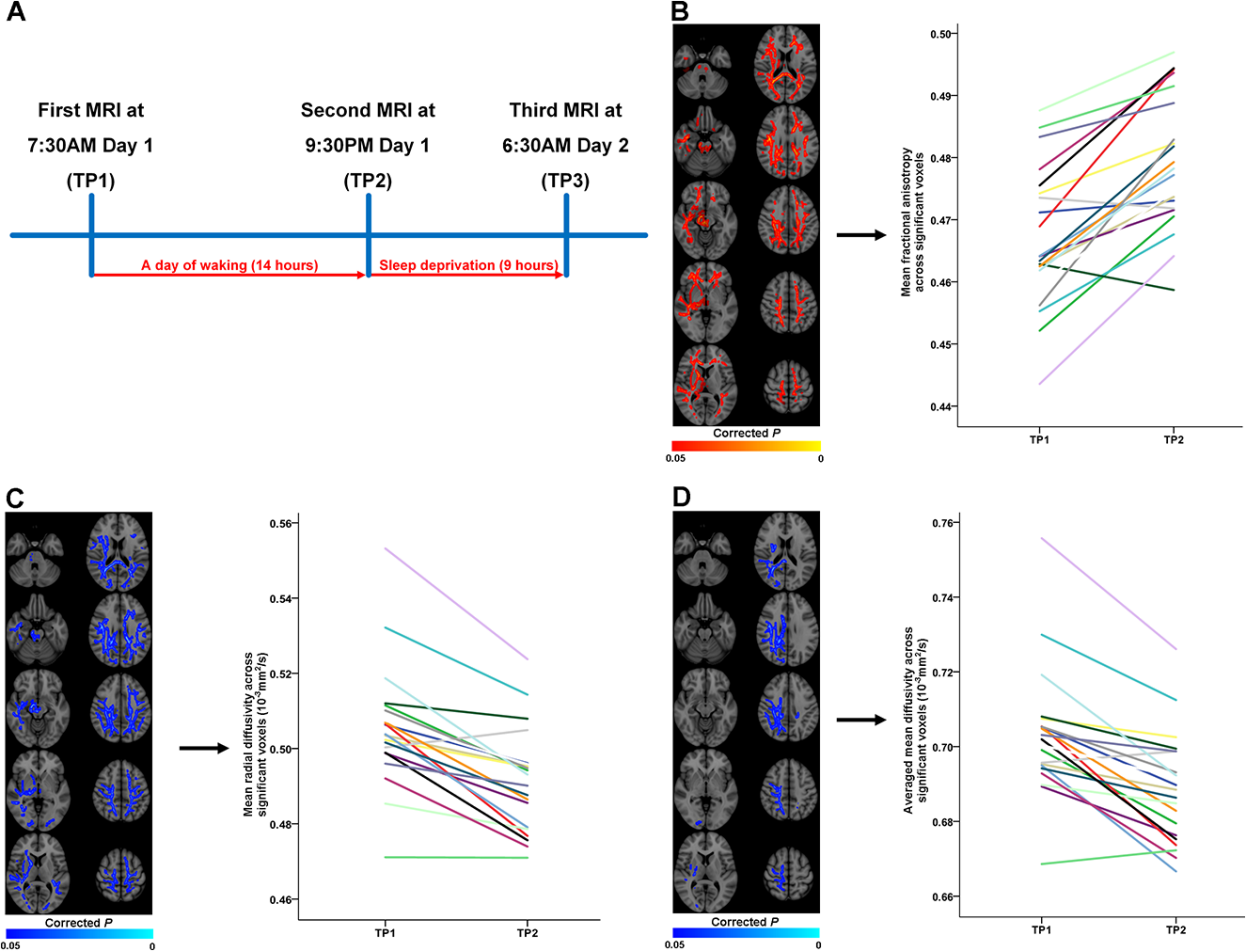
Changes in diffusion tensor imaging (DTI) indices of white matter microstructure after waking. (**A**) The participants underwent magnetic resonance imaging in the morning [7:30AM; time point (TP)1] after a night of normal sleep in their own homes, after a day of waking (TP2), and then after another 9 hours of waking (TP3). (**B**) Significant increases in fractional anisotropy (FA) after a day of waking (red-yellow color; left panel). (**C**) Significant decreases in radial diffusivity (RD) after a day of waking (blue colors; left panel). (**D**) Significant decreases in mean diffusivity (MD) after a day of waking (blue colors; left panel).

The participants underwent MRI in the morning [7:30AM; time point (TP)1] after a night of normal sleep in their own homes, after 14 hours of waking (TP2), and then after another 9 hours of waking (TP3; Fig 1A). No intake of caffeine, nicotine, or alcohol was allowed from the night before the study day until study completion and no intake of food or energy-containing fluids was allowed the 3 hours before each MRI session. Otherwise, no restrictions were placed on fluid intake before or during study participation. Participants were free to leave the hospital after the first MRI session, were instructed not to sleep and to refrain from physical activity, and returned at 9PM the same evening for the second MRI session. After the evening examination, the subjects stayed overnight at the hospital and were monitored by a research assistant to ensure that none fell asleep. Subjects were allowed to listen to music or a radio channel of their choosing during the MRI sessions. To prevent the subjects from falling asleep during the MRI, the participants completed a subtraction task that required them to press a button at an interval of a few seconds while in the MRI scanner. Each time the button was pressed, a signal was sent to the research assistant, which thereby monitored that the participants were awake. One subject fell asleep during the last two minutes of the DTI sequence at TP3.

Averaged DTI values at TP1 and TP2 across significant voxels are shown for each participant using individual colors in the right panels of (**B**)–(**D**). Values from the same participant are connected with a line. The left side of the brain images represents the right hemisphere.

### Assessment of Hydration State

No gold standard exists for the assessment of hydration state; however, information from two or more hydration indices is recommended for the evaluation of body hydration [26]. In the present study, body weight was measured immediately before imaging and blood samples were drawn immediately after each MRI session for the analysis of plasma osmolality and hematocrit. These indices of hydration were adjusted for in the analyses of changes in DTI parameters after waking. Hematocrit data was missing for one subject at TP1 and for another subject at TP3.

### Assessment of Head Motion

Head motion during DTI can affect anisotropy and diffusivity measurements [27, 28]. Here, we quantified four head motion measures for each scan, as described by Yendiki *et al*. [27]. First, we estimated *average volume-by-volume translation* using the translation component of the affine registration from each volume to the first volume. We computed the translation vector between each pair of consecutive volumes and averaged the magnitude of these translation vectors over all volumes in the scan. Second, we estimated *average volume-by-volume rotation* using the rotation component of the affine registration from each volume to the first volume. We computed the rotation angles between each pair of consecutive volumes and averaged the sum of the absolute values of these rotation angles over all volumes in the scan. Third, we estimated the *percentage of slices with signal drop-out* by computing the signal drop-out score proposed in Benner *et al*. for each slice in each volume, where slices with a score greater than 1 are considered to have suspect signal drop-out [28]. The percentage of slices in the entire scan that had a score greater than 1 were computed. Fourth, we estimated *signal drop-out severity* by computing the average signal drop-out score over all slices in the scan that had a score greater than 1. The head motion measures can be found in the S1 Dataset. Sixty-two out of the 63 scans had zero percentage of slices with signal drop-out. Thus, we adjusted the analyses of changes in DTI parameters after waking for average volume-by-volume translation and rotation.

### Assessment of Sleepiness

Sleepiness was assessed at TP3 using the Stanford Sleepiness Scale (SSS), i.e., a seven-point rating scale of subjective sleepiness sensitive to sleep deprivation where larger score indicates greater sleepiness [29]. Participants were allowed to report sleepiness using 0.5-scores.

### MRI Acquisition

Imaging was performed on a 3T Philips Achieva scanner (Philips Healthcare, Best, the Netherlands) using an 8-channel SENSE head coil (InVivo, Gainsville, Florida). For DTI, a fat-suppressed single-shot spin-echo echo-planar-imaging pulse sequence with 32 spatially independent diffusion-sensitized gradient directions was used with the following parameters: repetition time/echo time = 10439 ms/54 ms, b-value = 1000 s/mm^2^, FOV = 224 x 224 mm^2^, matrix = 112, reconstructed voxel size = 2 × 2 × 2 mm^3^, SENSE factor 2, 60 axial slices. The acquisition time was 7 min 40 s. A high-resolution 3D inversion recovery image set was also acquired for visualization purposes.

### MRI Analysis

All datasets were processed and analyzed at the multimodal imaging analysis lab at the Norwegian Centre for Mental Disorders Research (NORMENT), Oslo University Hospital. The image analysis was performed using the Functional Magnetic Resonance Imaging of the Brain (FMRIB) Software Library (FSL) [30]. Each DTI volume was affine registered to the b = 0 volume using the FMRIB's Linear Image Registration Tool (FLIRT) [31], correcting for intra-scan subject motion and eddy-current distortions. After removing non-brain tissue [32], voxel-wise eigenvalues and eigenvectors were extracted from the estimated diffusion tensor and FA was calculated. Mean diffusivity (MD) was defined as the mean of all three eigenvalues [(λ_1_ + λ_2_ + λ_3_)/3], AD as equal to the principal eigenvalue λ_1_, and RD as the mean of the second and third eigenvalues [(λ_2_ + λ_3_)/2]. Next, all individuals’ FA volumes were brought into standard space and skeletonized as performed by tract-based spatial statistics (TBSS) [33]. Briefly, all volumes were warped to the FMRIB58_FA template using local deformation procedures performed by FMRIB's Non-linear Image Registration Tool (FNIRT). A mean FA volume for all subjects was generated in standard space and thinned to create a mean FA skeleton. We thresholded and binarized the mean skeleton at FA > 0.2 to reduce the likelihood of partial volume effects, yielding a mask of 123.292 voxels. Each individuals’ FA maps were warped onto this skeleton mask by searching perpendicular from the skeleton for maximum values. Using maximum FA from the centers of the tracts further minimizes partial volume effects [33]. The resulting skeletons for each participant were fed into permutation-based cross-subject statistics. Similar warping and analyses were performed on the eigenvalue data.

### Statistical Analyses

Voxel wise analyses were performed using non-parametric permutation-based statistics [34] as implemented in the Randomise tool in FSL. First, individual difference skeleton maps were computed by subtracting the map obtained at one time point from the map obtained at another time point, thereby producing maps representing the difference in the various DTI parameter between TP1 and TP2, between TP2 and TP3, and between TP1 and TP3, respectively. Next, voxel-wise one-sample *t*-tests were performed testing for each voxel whether mean difference across subjects differed from zero. Threshold-free cluster enhancement [35] was used for inference and 5000 permutations were performed for each contrast. Statistical maps were thresholded at *P* < 0.05, fully corrected for multiple comparisons across space.

Averaged values of DTI measures in clusters showing significant changes after a day of waking (TP1 compared with TP2), after sleep deprivation (TP2 compared with TP3), and after 23 hours of waking (TP1 compared with TP3) were computed. These values were further examined in SPSS, version 18.0 for Windows (SPSS, Chicago, Illinois) and a two-tailed *P* value of < 0.05 was considered statistically significant. The relationship between significant changes in DTI measures after sleep deprivation (TP2 compared with TP3) and SSS scores at TP3 was examined using Pearson correlation tests. Furthermore, linear mixed models for repeated measurements were employed to adjust for potential effects of hydration indices and head motion measures on changes in the DTI parameters after waking. The analyses were rerun without the subject that fell asleep during the last two minutes of the DTI sequence at TP3; all findings remained significant after excluding this subject.

## Results

### DTI Changes After a Day of Waking

A day of waking (TP1 compared with TP2) was associated with widespread FA increases mainly involving right frontotemporal, right parieto-occipital, left frontal, and left parieto-occipital WM, the corpus callosum, the thalamus, and the brain stem (Fig 1B and Table 1). Nineteen of the 21 participants showed increased FA in these clusters after waking (2.7% mean increase across significant clusters). There were no areas with decreased FA. FA increases were mainly driven by anatomically overlapping decreases in RD (2.8% mean decrease; Fig 1C). Twenty of the subjects exhibited reduced RD in these voxels. Decreases in MD were also found, mainly in right parieto-occipital WM (Fig 1D). Nineteen of the subjects showed reduced MD in these clusters after waking (2.1% mean decrease across significant clusters). No significant changes in AD were observed. All DTI changes after a day of waking remained significant after adjusting for hydration (linear mixed models; *P* < 0.0001, *P* < 0.001, and *P* < 0.001 for FA, RD, and MD, respectively), average volume-by-volume translation (linear mixed models; *P* < 0.00001, *P* < 0.00001, and *P* < 0.00001 for FA, RD, and MD) and rotation (linear mixed models; *P* = 0.000001, *P* < 0.0000001, and *P* < 0.00001 for FA, RD, and MD).

**Table 1:** Clusters with significant changes in DTI indices of white matter microstructure after a day of waking (TP1 compared with TP2).

DTI parameter	No. of voxels in cluster	Change after a day of waking	MNI (x, y, z) maxima	Anatomical region of the peak voxel^a^	Peak voxel *P*-value
FA	21062	↑	39, −8, 27	R SLF	0.006
	9273	↑	−23, 31, 19	L ATR, IFOF, UF	0.024
	175	↑	−8, −37, 56	L Cingulum	0.047
	64	↑	−18, 11, −25	L Orbitofrontal*	0.046
	11	↑	−15, −39, 63	L CST	0.049
	2	↑	−18, −36, 62	L CST	0.049
RD	30466	↓	34, −29, 37	R SLF	<0.001
MD	9516	↓	28, −57, 19	R IFOF, ILF, Fmaj	0.005
	62	↓	−19, −37, 37	L Cingulum	0.048
	42	↓	−19, −54, 43	L Precuneus*	0.048
	10	↓	45, −18, 45	R Postcentral gyrus*	0.048
	2	↓	33, −47, 29	R SLF, ILF	0.049
	1	↓	34, −48, 26	R SLF, ILF	0.049

DTI; diffusion tensor imaging. TP; time point. MNI; Montreal Neurological Institute. R; right. L; left. SLF; superior longitudinal fasciculus. ATR; anterior thalamic radiation. IFOF; inferior fronto-occipital fasciculus. UF; uncinate fasciculus. CST; cortico-spinal tract. Fmaj; forceps major. ILF; inferior longitudinal fasciculus.

^a^Anatomical region based on Johns Hopkins University (JHU) white matter tractography atlas and the ICBM-DTI-81 white matter labels atlas [36–38].

*Not within the white matter atlases; gross anatomical description.

### DTI Changes After Sleep Deprivation

**Fig 2:**
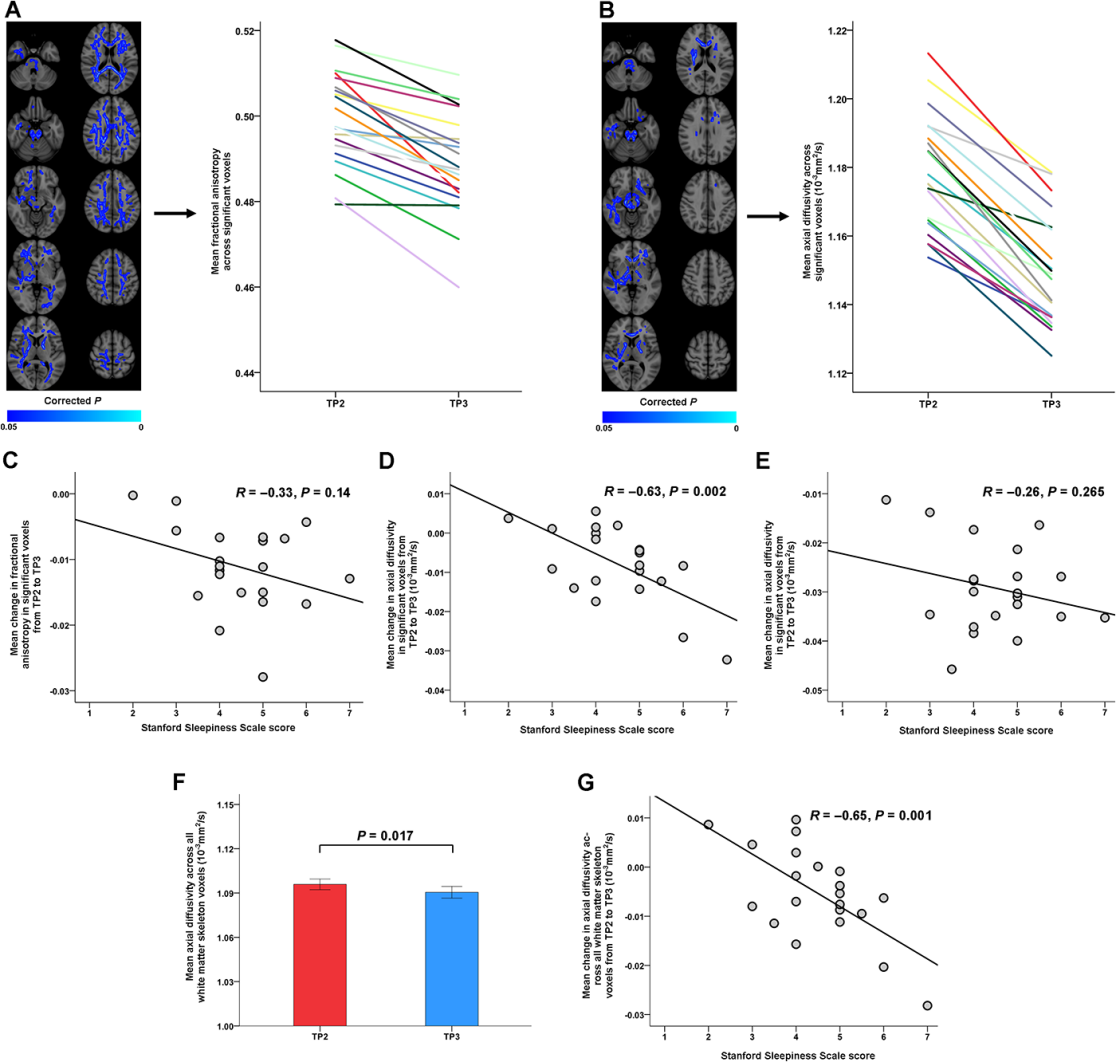
Changes in diffusion tensor imaging (DTI) indices of white matter microstructure after sleep deprivation and associations with sleepiness. (**A**) Significant decreases in fractional anisotropy (FA) after sleep deprivation (blue colors; left panel). (**B**) Significant decreases in axial diffusivity (AD) after sleep deprivation (blue colors; left panel). Averaged DTI values at time point (TP)2 and TP3 across significant voxels are shown for each participant using individual colors in the right panels of (**A**) and (**B**). Values from the same participant are connected with a line. (**C**) No significant relationship was observed between the decrease in FA in the voxels shown in (**A**) and Stanford Sleepiness Scale (SSS) score at TP3 (*R* = −0.33, *P* = 0.14). (**D**) Because the FA decreases in the significant voxels of (**A**) were mainly driven by AD reductions, we examined whether reductions in averaged AD within these clusters correlated with SSS score and found a significant negative association (*R* = −0.63, *P* = 0.002), indicating greater sleepiness in subjects with larger AD reductions after sleep deprivation. (**E**) No significant relationship was found between AD reductions across the voxels shown in (**B**) and SSS score (*R* = −0.26, *P* = 0.265). (**F,G**) Averaged AD across all voxels of the white matter skeleton decreased significantly from TP2 to TP3; this decrease was significantly correlated with sleepiness at TP3 (*R* = −0.65, *P* = 0.001). The left side of the brain images represents the right hemisphere.

Sleep deprivation (TP2 compared with TP3) was associated with widespread FA decreases, mainly including bilateral frontotemporal and parieto-occipital WM, the corpus callosum, the thalamus, and the brain stem (Fig 2A and Table 2). All subjects showed decreased FA across these clusters after sleep deprivation (2.2% mean decrease across significant clusters). There were no areas with increased FA. Notably, reductions in FA were mainly driven by overlapping decreases in AD (Fig 2B). AD decreases across these clusters were found in all subjects (2.5% mean decrease). No significant RD or MD changes were observed after sleep deprivation. Changes in DTI indices after sleep deprivation remained significant after correcting for hydration (linear mixed models; *P* < 0.00001 and *P* < 0.000001 for FA and AD, respectively), average volume-by-volume translation (linear mixed models; *P* < 0.0000001 and *P* < 0.0000001 for FA and AD) and rotation (linear mixed models; *P* < 0.0000001 and *P* < 0.0000001 for FA and AD).

**Table 2:** Clusters with significant changes in DTI indices of white matter microstructure after sleep deprivation (TP2 compared with TP3).

DTI parameter	No. of voxels in cluster	Change after sleep deprivation	MNI (x, y, z) maxima	Anatomical region of the peak voxel^a^	Peak voxel *P*-value
FA	30337	↓	21, −41, 27	R Splenium*	0.003
AD	7680	↓	17, −4, 5	R PLIC	0.021
	2676	↓	−1, 29, 8	Fmin	0.029
	551	↓	−20, 11, −23	L Orbitofrontal*	0.043
	12	↓	10, −22, 7	R ATR	0.049
	7	↓	−17, 36, 18	L Cingulum, Fmin	0.049
	4	↓	19, 28, 35	R Superior frontal gyrus*	0.049

DTI; diffusion tensor imaging. TP; time point. MNI; Montreal Neurological Institute. R; right. L; left. PLIC; posterior limb of internal capsule. Fmin; forceps minor. ATR; anterior thalamic radiation.

^a^Anatomical region based on Johns Hopkins University (JHU) white matter tractography atlas and the ICBM-DTI-81 white matter labels atlas [36–38].

*Not within the white matter atlases; gross anatomical description.

### Relationship between DTI Changes and Sleepiness After Sleep Deprivation

We then examined whether larger decreases in FA and AD after sleep deprivation (TP2 compared with TP3) were related to greater sleepiness at TP3, as measured by the SSS. No significant relationship was observed between the decrease in FA in the clusters shown in Fig 2A and SSS at TP3 (*R* = −0.33, *P* = 0.14; Fig 2C). Because the FA decreases were mainly driven by AD reductions, we examined whether reductions in averaged AD within the same clusters correlated with sleepiness. This analysis revealed a significant negative association (*R* = −0.63, *P* = 0.002; Fig 2D), indicating greater sleepiness in subjects with larger AD reductions after sleep deprivation. However, no significant relationship was found between mean AD reduction across the clusters shown in Fig 2B and sleepiness (*R* = −0.26, *P* = 0.265; Fig 2E). We therefore further explored the AD changes after sleep deprivation and found that AD averaged across all voxels of the WM skeleton decreased significantly from TP2 to TP3 (*P* = 0.017, *P* = 0.002, *P* = 0.017, and *P* = 0.019 before and after adjusting for the hydration indices, average volume-by-volume translation, and average volume-by-volume rotation using linear mixed models, respectively; Fig 2F). The decrease in AD across all skeleton voxels was significantly associated with sleepiness (*R* = −0.65, *P* = 0.001; Fig 2G).

### DTI Changes After 23 Hours of Waking

Significant reductions in AD, RD, and MD were also found when TP1 and TP3 were compared (S1 Fig and S1 Table). AD was mainly decreased in right frontotemporal and right parieto-occipital WM, the corpus callosum, and the brain stem. All subjects showed decreased AD across these clusters after 23 hours of waking (2.2% mean decrease across significant clusters). RD was decreased in clusters involving right parieto-occipital WM and twenty of the subjects exhibited reduced RD in these voxels (2.8% mean decrease across significant clusters). MD was reduced in clusters mainly involving right frontotemporal and right parieto-occipital WM and the brain stem. All subjects showed decreased MD across these clusters after 23 hours of waking (2.3% mean decrease across significant clusters). No significant FA change was observed when TP1 and TP3 were compared. The DTI changes after 23 hours of waking remained significant after adjusting for hydration (linear mixed models; *P* < 0.000001, *P* < 0.000001, and *P* < 0.000001 for AD, RD, and MD, respectively), average volume-by-volume translation (linear mixed models; *P* < 0.0000001, *P* < 0.0000001, and *P* < 0.0000001 for AD, RD, and MD) and rotation (linear mixed models; *P* = 0.0000001, *P* < 0.0000001, and *P* < 0.0000001 for AD, RD, and MD).

## Discussion

This is, to our knowledge, the first study to examine the effects of sleep deprivation on DTI indices of WM microstructure. We found that a day of waking followed by sleep deprivation was associated with a sequential pattern of widespread changes in DTI indices of cerebral WM microstructure. Specifically, we observed widespread FA increases after a day of waking, which were mainly driven by RD reductions, and widespread FA decreases after sleep deprivation, which were mainly explained by reductions in AD. In addition, larger decreases in AD were found in subjects with greater sleepiness after sleep deprivation. Together, these findings indicate that human brain WM exhibits circadian plasticity and susceptibility to insufficient sleep.

Although the waking-related DTI changes were highly consistent across participants, replication of widespread WM alterations within hours of waking is needed. In addition, the present findings must be interpreted in light of several important limitations. First, the biological substrate for the DTI changes observed after waking remains unknown. Histological studies are needed to clarify the microanatomical substrate for waking-related changes in DTI parameters. Second, we did not examine whether waking-related alterations in WM microstructure reverse after subsequent sleep. Third, we did not control for chronotype and the participants might have been in different circadian phases at the time of measurements. Fourth, the amount of microsleep among the participants was not assessed. Furthermore, we cannot rule out the possibility that subjects slept between the first and second MRI session; however, this would, at least in theory, attenuate the waking-related DTI changes, rather than inflate them. Nevertheless, future studies may adapt a design with more rigorous control over the subjects’ sleep-wake cycle, e.g., by housing subjects in a sleep laboratory and by continuously recording the electroencephalogram (EEG). Finally, future studies should examine the relationship between waking-related alterations in DTI indices and EEG changes, such as delta and theta power increases, and functional measures, including objective estimates of sleepiness and neuropsychological functioning.

The waking-related DTI changes were widespread, involved most of the major WM tracts, and included regions within the brain stem. The latter finding is noteworthy, given the importance of brain stem nuclei for wakefulness [11, 39]. Specifically, the ascending arousal system, which is crucial for cortical and thalamic activation during waking, involves several cell groups in the upper brain stem, including pedunculopontine, laterodorsal tegmental, and monoaminergic nuclei [11, 39]. Thus, it can be hypothesized that altered WM microstructure, particularly within the ascending arousal system, is a neurobiological substrate for sleepiness. Interestingly, and in support of this hypothesis, we found that subjects exhibiting larger reductions in AD had greater subjective sleepiness after sleep deprivation. A link between vulnerability to insufficient sleep and WM microstructure is also supported by two previous cross-sectional DTI studies [40, 41]. Rocklage *et al*. reported that individuals susceptible to sleep deprivation had lower FA values than less susceptible subjects in multiple brain WM regions [40]. In support of these findings, Cui *et al*. observed that higher FA in left frontoparietal WM connections predicted better resistance to sleep deprivation [41].

Among the other regions that exhibited alterations in DTI parameters after a day of waking and sleep deprivation were areas within the frontal lobes. These findings are consistent with previous studies indicating that the frontal lobes are susceptible to lack of sleep. For example, several research groups have reported reduced metabolism in frontal regions after sleep deprivation [42, 43]. In addition, neuropsychological [44, 45] and electrophysiological [46–49] studies indicate that, in humans, changes in the sleep-wake cycle may be more prominent in the prefrontal cortices than in other cortical areas. Furthermore, the best established markers of increased sleep pressure are increased frontal theta band power in the waking EEG and increased frontal delta band power in the sleep EEG [46–49].

There were significant reductions in AD, RD, and MD, but not in FA, when the two morning examinations were compared. FA is a function of the ratio between AD and RD [19, 20]. Thus, a simultaneous reduction in both AD and RD may result in no change in FA, as shown previously [50]. The fact that both AD and RD were reduced likely explains why no significant changes in FA were found between the two morning examinations. We also found less extensive changes in the other DTI parameters when the two morning examinations were compared. This suggests that the circadian rhythm, in addition to the cumulative effects of waking, might influence DTI indices of WM microstructure. Although there is a scarcity of longitudinal brain DTI studies during the sleep-wake cycle, a recent study provided evidence for a link between WM microstructure and circadian regulation. Here, Rosenberg *et al*. reported that individuals with an early chronotype, i.e., subjects which tend to wake up early in the morning and prefer to go to bed early in the evening, had higher FA and lower MD than subjects with a late chronotype, mainly in left frontal lobe WM [51].

We observed widespread RD decreases, but no AD changes after a day of waking, whereas sleep deprivation was associated with AD reductions and no RD alterations. These findings raise the possibility that physiological waking length and sleep deprivation are associated with qualitatively distinct changes in WM microstructure. However, DTI parameters are influenced by a number of tissue properties, including myelin structure, axonal membrane permeability, axonal diameter, astrocytic cell processes, and tissue perfusion [19, 52]. Consequently, neurobiological interpretations of the waking-related DTI changes observed in the present study should be made with caution. Notwithstanding this limitation, it has been shown that RD is sensitive to myelin alterations and that AD can reflect axonal integrity [19, 53, 54]. Moreover, recent research indicates that sleep is important for myelin and cell membrane maintenance in the brain and that these structures might be particularly susceptible to insufficient sleep [17, 18]. Thus, although speculative, the DTI changes observed in the present study might be related to waking-related structural alterations in WM myelin and axonal membranes.

Another mechanism that could explain the present findings is reduced interstitial space volume and increased resistance to water flux in the brain after waking than during sleep, as recently observed in mice [55]. Changes in body hydration could also lead to alterations in DTI parameters. However, the waking-related DTI changes remained highly significant after adjusting for the hydration indices. Thus, it is unlikely that changes in body hydration underlie the findings of the present study.

The findings of the present study are consistent with the emerging view that structural changes can be observed in the adult brain over hours to days. Hofstetter *et al*. demonstrated significant MD and AD decreases in the fornix of adult humans and rats after 2 hours and a day of spatial learning, respectively [56]. In addition, the researchers found significant reductions in hippocampal MD after spatial learning and that the DTI changes in the fornix and the hippocampus were highly correlated in both species [56, 57]. This suggests that rapid alterations in WM microstructure can be accompanied by changes in corresponding gray matter (GM). Supporting the notion that structural GM changes can occur within brief periods of time, Draganski *et al*. showed in a seminal study that learning a cascade juggling task over a three-month period was associated with increased GM density in the occipito-temporal cortex [58]. Importantly, they later replicated this finding and demonstrated GM increases after 7 days of juggling training [59]. More recently, Tost *et al*. observed a volumetric decrease in the ventral putamen of healthy volunteers 1–2 hours after haloperidol infusion; this was partially reversed approximately 24 hours after drug administration [60]. Taken together, these studies indicate that alterations in both white and gray matter structure can occur within hours in the adult brain. Whether waking-related structural alterations are specific for WM or also occur in GM should be examined in further studies.

The prospect of circadian WM plasticity is intriguing and has, if confirmed by other studies, implications for future research. First, histological studies should be conducted to elucidate the microanatomical substrate for waking-related changes in DTI parameters. Clarifying the biological underpinnings of these alterations could significantly advance our understanding of the neurobiological effects of waking and sleep. Second, future studies could examine whether waking-related DTI changes are experience-dependent or merely reflect nonspecific effects of cumulative wakefulness. Third, sleep deprivation results in rapid antidepressive response within 24 hours in approximately 60% of subjects with unipolar and bipolar depression, yet the mechanisms underlying this effect remain incompletely understood [8, 9]. In addition, WM alterations have been consistently identified in bipolar disorders [61, 62] and in several [63, 64], but not all [65] studies of unipolar depression using DTI. Therefore, future studies could test the hypothesis that waking-related changes in WM microstructure contribute to antidepressive effects of sleep deprivation. The potential link between sleep deprivation-related antidepressive response and WM microstructure is supported by the findings of Bollettini *et al*. [66]. They examined brain DTI indices in depressed individuals with bipolar disorder before repeated sleep deprivation and morning light therapy and found that higher RD and MD in right hemisphere WM tracts were associated with reduced antidepressive response. Fourth, it has been shown that circadian rhythm and sensitivity to sleep deprivation are influenced by variants in circadian clock genes [3, 67]. Whether circadian clock genes modify waking-related changes in DTI parameters is therefore another potential research avenue. Finally, the present study and previous findings indicate that widespread changes in WM DTI indices can occur within hours of waking [22]. Future studies should therefore consider the possibility that time of the day might confound group analyses of DTI parameters.

Taken together, our results indicate that human brain WM exhibits circadian plasticity and susceptibility to insufficient sleep. The waking-related DTI changes may be related to alterations in WM myelin and axonal membranes, yet histological studies are required to elucidate the precise underlying microanatomical substrate. In addition, further studies are needed to clarify whether changes in WM microstructure serve as neurobiological substrates of sleepiness.

## Supporting Information

S1 Dataset Data underlying the findings of the study. (SAV)

S1 Fig Changes in diffusion tensor imaging indices of white matter microstructure after 23 hours of waking. (**A**) Significant decreases in axial diffusivity after 23 hours of waking (blue colors; left panel). (**B**) Significant decreases in radial diffusivity after 23 hours of waking (blue colors; left panel). (**C**) Significant decreases in mean diffusivity after 23 hours of waking (blue colors; left panel). Averaged DTI values at time point (TP)1 and TP3 across significant voxels are shown for each participant using individual colors in the right panels of (**A**)–(**C**). Values from the same participant are connected with a line. The left side of the brain images represents the right hemisphere.
(TIF)

S1 Table Clusters with significant changes in diffusion tensor imaging indices of white matter microstructure after 23 hours of waking. (DOC)

## References

[1] Walker MP, Stickgold R. Sleep, memory, and plasticity. Annu Rev Psychol. 2006;57: 139–166. 16318592 10.1146/annurev.psych.56.091103.070307

[2] Luyster FS, Strollo PJ Jr, Zee PC, Walsh JK. Sleep: a health imperative. Sleep. 2012;35: 727–734. 10.5665/sleep.1846 22654183 PMC3353049

[3] Cirelli C. The genetic and molecular regulation of sleep: from fruit flies to humans. Nat Rev Neurosci. 2009;10: 549–560. 10.1038/nrn2683 19617891 PMC2767184

[4] Killgore WD. Effects of sleep deprivation on cognition. Prog Brain Res. 2010;185: 105–129. 10.1016/B978-0-444-53702-7.00007-5 21075236

[5] Lim J, Dinges DF. A meta-analysis of the impact of short-term sleep deprivation on cognitive variables. Psychol Bull. 2010;136: 375–389. 10.1037/a0018883 20438143 PMC3290659

[6] Wulff K, Gatti S, Wettstein JG, Foster RG. Sleep and circadian rhythm disruption in psychiatric and neurodegenerative disease. Nat Rev Neurosci. 2010;11: 589–599. 10.1038/nrn2868 20631712

[7] McClung CA. How might circadian rhythms control mood? Let me count the ways. Biol Psychiatry. 2013;74: 242–249. 10.1016/j.biopsych.2013.02.019 23558300 PMC3725187

[8] Benedetti F, Colombo C. Sleep deprivation in mood disorders. Neuropsychobiology. 2011;64: 141–151. 10.1159/000328947 21811084

[9] Wu JC, Bunney WE. The biological basis of an antidepressant response to sleep deprivation and relapse: review and hypothesis. Am J Psychiatry. 1990;147: 14–21. 2403471 10.1176/ajp.147.1.14

[10] Oswald I. Human brain protein, drugs and dreams. Nature. 1969;223: 893–897. 4308512 10.1038/223893a0

[11] Saper CB, Fuller PM, Pedersen NP, Lu J, Scammell TE. Sleep state switching. Neuron. 2010;68: 1023–1042. 10.1016/j.neuron.2010.11.032 21172606 PMC3026325

[12] Mackiewicz M, Shockley KR, Romer MA, Galante RJ, Zimmerman JE, Naidoo N, et al Macromolecule biosynthesis: a key function of sleep. Physiol Genomics. 2007;31: 441–457. 17698924 10.1152/physiolgenomics.00275.2006

[13] Mackiewicz M, Zimmerman JE, Shockley KR, Churchill GA, Pack AI. What are microarrays teaching us about sleep? Trends Mol Med. 2009;15: 79–87. 10.1016/j.molmed.2008.12.002 19162550 PMC2942088

[14] Mongrain V, Hernandez SA, Pradervand S, Dorsaz S, Curie T, Hagiwara G, et al Separating the contribution of glucocorticoids and wakefulness to the molecular and electrophysiological correlates of sleep homeostasis. Sleep. 2010;33: 1147–1157. 20857860 10.1093/sleep/33.9.1147 PMC2938796

[15] Cirelli C, LaVaute TM, Tononi G. Sleep and wakefulness modulate gene expression in Drosophila. J Neurochem. 2005;94: 1411–1419. 16001966 10.1111/j.1471-4159.2005.03291.x

[16] Cirelli C, Gutierrez CM, Tononi G. Extensive and divergent effects of sleep and wakefulness on brain gene expression. Neuron. 2004;41: 35–43. 14715133 10.1016/s0896-6273(03)00814-6

[17] Bellesi M, Pfister-Genskow M, Maret S, Keles S, Tononi G, Cirelli C. Effects of sleep and wake on oligodendrocytes and their precursors. J Neurosci. 2013;33: 14288–14300. 10.1523/JNEUROSCI.5102-12.2013 24005282 PMC3874087

[18] Hinard V, Mikhail C, Pradervand S, Curie T, Houtkooper RH, Auwerx J, et al Key electrophysiological, molecular, and metabolic signatures of sleep and wakefulness revealed in primary cortical cultures. J Neurosci. 2012;32: 12506–12517. 10.1523/JNEUROSCI.2306-12.2012 22956841 PMC6621272

[19] Beaulieu C. The basis of anisotropic water diffusion in the nervous system—a technical review. NMR Biomed. 2002;15: 435–455. 12489094 10.1002/nbm.782

[20] Mori S, Zhang J. Principles of diffusion tensor imaging and its applications to basic neuroscience research. Neuron. 2006;51: 527–539. 16950152 10.1016/j.neuron.2006.08.012

[21] Basser PJ, Mattiello J, LeBihan D. Estimation of the effective self-diffusion tensor from the NMR spin echo. J Magn Reson B. 1994;103: 247–254. 8019776 10.1006/jmrb.1994.1037

[22] Jiang C, Zhang L, Zou C, Long X, Liu X, Zheng H, et al Diurnal microstructural variations in healthy adult brain revealed by diffusion tensor imaging. PLoS One. 2014;9: e84822 10.1371/journal.pone.0084822 24400118 PMC3882241

[23] Shannon BJ, Dosenbach RA, Su Y, Vlassenko AG, Larson-Prior LJ, Nolan TS, et al Morning-evening variation in human brain metabolism and memory circuits. J Neurophysiol. 2013;109: 1444–1456. 10.1152/jn.00651.2012 23197455 PMC3602835

[24] De Havas JA, Parimal S, Soon CS, Chee MW. Sleep deprivation reduces default mode network connectivity and anti-correlation during rest and task performance. NeuroImage. 2012;59: 1745–1751. 10.1016/j.neuroimage.2011.08.026 21872664

[25] Samann PG, Tully C, Spoormaker VI, Wetter TC, Holsboer F, Wehrle R, et al Increased sleep pressure reduces resting state functional connectivity. Magma. 2010;23: 375–389. 10.1007/s10334-010-0213-z 20473549

[26] Armstrong LE. Assessing hydration status: the elusive gold standard. J Am Coll Nutr. 2007;26: 575S–584S. 17921468 10.1080/07315724.2007.10719661

[27] Yendiki A, Koldewyn K, Kakunoori S, Kanwisher N, Fischl B. Spurious group differences due to head motion in a diffusion MRI study. NeuroImage. 2013;88c: 79–90. 10.1016/j.neuroimage.2013.11.027 24269273 PMC4029882

[28] Benner T, van der Kouwe AJ, Sorensen AG. Diffusion imaging with prospective motion correction and reacquisition. Magn Reson Med. 2011;66: 154–167. 10.1002/mrm.22837 21695721 PMC3121006

[29] Hoddes E, Zarcone V, Smythe H, Phillips R, Dement WC. Quantification of sleepiness: a new approach. Psychophysiology. 1973;10: 431–436. 4719486 10.1111/j.1469-8986.1973.tb00801.x

[30] Smith SM, Jenkinson M, Woolrich MW, Beckmann CF, Behrens TE, Johansen-Berg H, et al Advances in functional and structural MR image analysis and implementation as FSL. NeuroImage. 2004;23 Suppl 1: S208–219. 15501092 10.1016/j.neuroimage.2004.07.051

[31] Jenkinson M, Smith S. A global optimisation method for robust affine registration of brain images. Med Image Anal. 2001;5: 143–156. 11516708 10.1016/s1361-8415(01)00036-6

[32] Smith SM. Fast robust automated brain extraction. Hum Brain Mapp. 2002;17: 143–155. 12391568 10.1002/hbm.10062 PMC6871816

[33] Smith SM, Jenkinson M, Johansen-Berg H, Rueckert D, Nichols TE, Mackay CE, et al Tract-based spatial statistics: voxelwise analysis of multi-subject diffusion data. NeuroImage. 2006;31: 1487–1505. 16624579 10.1016/j.neuroimage.2006.02.024

[34] Nichols TE, Holmes AP. Nonparametric permutation tests for functional neuroimaging: a primer with examples. Hum Brain Mapp. 2002;15: 1–25. 11747097 10.1002/hbm.1058 PMC6871862

[35] Kempton MJ, Ettinger U, Schmechtig A, Winter EM, Smith L, McMorris T, et al Effects of acute dehydration on brain morphology in healthy humans. Hum Brain Mapp. 2009;30: 291–298. 18064587 10.1002/hbm.20500 PMC6871128

[36] Hua K, Zhang J, Wakana S, Jiang H, Li X, Reich DS, et al Tract probability maps in stereotaxic spaces: analyses of white matter anatomy and tract-specific quantification. NeuroImage. 2008;39: 336–347. 17931890 10.1016/j.neuroimage.2007.07.053 PMC2724595

[37] Wakana S, Jiang H, Nagae-Poetscher LM, van Zijl PC, Mori S. Fiber tract-based atlas of human white matter anatomy. Radiology. 2004;230: 77–87. 14645885 10.1148/radiol.2301021640

[38] Mori S, Wakana S, Van Zijl PC, Nagae-Poetscher LM. MRI Atlas of Human White Matter Amsterdam: Elsevier; 2005.

[39] Saper CB, Scammell TE, Lu J. Hypothalamic regulation of sleep and circadian rhythms. Nature. 2005;437: 1257–1263. 16251950 10.1038/nature04284

[40] Rocklage M, Williams V, Pacheco J, Schnyer DM. White matter differences predict cognitive vulnerability to sleep deprivation. Sleep. 2009;32: 1100–1103. 19725262 10.1093/sleep/32.8.1100 PMC2717201

[41] Cui J, Tkachenko O, Gogel H, Kipman M, Preer LA, Weber M, et al Microstructure of frontoparietal connections predicts individual resistance to sleep deprivation. NeuroImage. 2015;106: 123–133. 10.1016/j.neuroimage.2014.11.035 25463450

[42] Thomas M, Sing H, Belenky G, Holcomb H, Mayberg H, Dannals R, et al Neural basis of alertness and cognitive performance impairments during sleepiness. I. Effects of 24 h of sleep deprivation on waking human regional brain activity. J Sleep Res. 2000;9: 335–352. 11123521 10.1046/j.1365-2869.2000.00225.x

[43] Wu JC, Gillin JC, Buchsbaum MS, Chen P, Keator DB, Khosla Wu N, et al Frontal lobe metabolic decreases with sleep deprivation not totally reversed by recovery sleep. Neuropsychopharmacology. 2006;31: 2783–2792. 16880772 10.1038/sj.npp.1301166

[44] Muzur A, Pace-Schott EF, Hobson JA. The prefrontal cortex in sleep. Trends Cogn Sci. 2002;6: 475–481. 12457899 10.1016/s1364-6613(02)01992-7

[45] Horne JA. Human sleep, sleep loss and behaviour. Implications for the prefrontal cortex and psychiatric disorder. Br J Psychiatry. 1993;162: 413–419. 8453439 10.1192/bjp.162.3.413

[46] Finelli LA, Baumann H, Borbely AA, Achermann P. Dual electroencephalogram markers of human sleep homeostasis: correlation between theta activity in waking and slow-wave activity in sleep. Neuroscience. 2000;101: 523–529. 11113301 10.1016/s0306-4522(00)00409-7

[47] Cajochen C, Knoblauch V, Krauchi K, Renz C, Wirz-Justice A. Dynamics of frontal EEG activity, sleepiness and body temperature under high and low sleep pressure. Neuroreport. 2001;12: 2277–2281. 11447349 10.1097/00001756-200107200-00046

[48] Tinguely G, Finelli LA, Landolt HP, Borbely AA, Achermann P. Functional EEG topography in sleep and waking: state-dependent and state-independent features. NeuroImage. 2006;32: 283–292. 16650779 10.1016/j.neuroimage.2006.03.017

[49] De Gennaro L, Marzano C, Veniero D, Moroni F, Fratello F, Curcio G, et al Neurophysiological correlates of sleepiness: a combined TMS and EEG study. NeuroImage. 2007;36: 1277–1287. 17524675 10.1016/j.neuroimage.2007.04.013

[50] Acosta-Cabronero J, Williams GB, Pengas G, Nestor PJ. Absolute diffusivities define the landscape of white matter degeneration in Alzheimer's disease. Brain. 2010;133: 529–539. 10.1093/brain/awp257 19914928

[51] Rosenberg J, Maximov II, Reske M, Grinberg F, Shah NJ. "Early to bed, early to rise": diffusion tensor imaging identifies chronotype-specificity. NeuroImage. 2014;84: 428–434. 10.1016/j.neuroimage.2013.07.086 24001455

[52] Zatorre RJ, Fields RD, Johansen-Berg H. Plasticity in gray and white: neuroimaging changes in brain structure during learning. Nat Neurosci. 2012;15: 528–536. 10.1038/nn.3045 22426254 PMC3660656

[53] Budde MD, Xie M, Cross AH, Song SK. Axial diffusivity is the primary correlate of axonal injury in the experimental autoimmune encephalomyelitis spinal cord: a quantitative pixelwise analysis. J Neurosci. 2009;29: 2805–2813. 10.1523/JNEUROSCI.4605-08.2009 19261876 PMC2673458

[54] Song SK, Sun SW, Ramsbottom MJ, Chang C, Russell J, Cross AH. Dysmyelination revealed through MRI as increased radial (but unchanged axial) diffusion of water. NeuroImage. 2002;17: 1429–1436. 12414282 10.1006/nimg.2002.1267

[55] Xie L, Kang H, Xu Q, Chen MJ, Liao Y, Thiyagarajan M, et al Sleep drives metabolite clearance from the adult brain. Science. 2013;342: 373–377. 10.1126/science.1241224 24136970 PMC3880190

[56] Hofstetter S, Tavor I, Tzur Moryosef S, Assaf Y. Short-term learning induces white matter plasticity in the fornix. J Neurosci. 2013;33: 12844–12850. 10.1523/JNEUROSCI.4520-12.2013 23904619 PMC6618548

[57] Sagi Y, Tavor I, Hofstetter S, Tzur-Moryosef S, Blumenfeld-Katzir T, Assaf Y. Learning in the fast lane: new insights into neuroplasticity. Neuron. 2012;73: 1195–1203. 10.1016/j.neuron.2012.01.025 22445346

[58] Draganski B, Gaser C, Busch V, Schuierer G, Bogdahn U, May A. Neuroplasticity: changes in grey matter induced by training. Nature. 2004;427: 311–312. 14737157 10.1038/427311a

[59] Driemeyer J, Boyke J, Gaser C, Buchel C, May A. Changes in gray matter induced by learning—revisited. PLoS One. 2008;3: e2669 10.1371/journal.pone.0002669 18648501 PMC2447176

[60] Tost H, Braus DF, Hakimi S, Ruf M, Vollmert C, Hohn F, et al Acute D2 receptor blockade induces rapid, reversible remodeling in human cortical-striatal circuits. Nat Neurosci. 2010;13: 920–922. 10.1038/nn.2572 20526332

[61] Nortje G, Stein DJ, Radua J, Mataix-Cols D, Horn N. Systematic review and voxel-based meta-analysis of diffusion tensor imaging studies in bipolar disorder. Journal of affective disorders. 2013;150: 192–200. 10.1016/j.jad.2013.05.034 23810479

[62] Vederine FE, Wessa M, Leboyer M, Houenou J. A meta-analysis of whole-brain diffusion tensor imaging studies in bipolar disorder. Prog Neuropsychopharmacol Biol Psychiatry. 2011;35: 1820–1826. 10.1016/j.pnpbp.2011.05.009 21624424

[63] Zou K, Huang X, Li T, Gong Q, Li Z, Ou-yang L, et al Alterations of white matter integrity in adults with major depressive disorder: a magnetic resonance imaging study. J Psychiatry Neurosci. 2008;33: 525–530. 18982175 PMC2575756

[64] Cole J, Chaddock CA, Farmer AE, Aitchison KJ, Simmons A, McGuffin P, et al White matter abnormalities and illness severity in major depressive disorder. Br J Psychiatry. 2012;201: 33–39. 10.1192/bjp.bp.111.100594 22576724

[65] Choi KS, Holtzheimer PE, Franco AR, Kelley ME, Dunlop BW, Hu XP, et al. Reconciling Variable Findings of White Matter Integrity in Major Depressive Disorder. Neuropsychopharmacology. 2013; e-pub ahead of print 19 December 2013; 10.1038/npp.2013.345 PMC3988550 24352368

[66] Bollettini I, Poletti S, Locatelli C, Vai B, Smeraldi E, Colombo C, et al Disruption of white matter integrity marks poor antidepressant response in bipolar disorder. Journal of affective disorders. 2015;174: 233–240. 10.1016/j.jad.2014.11.010 25527993

[67] Takahashi JS, Hong HK, Ko CH, McDearmon EL. The genetics of mammalian circadian order and disorder: implications for physiology and disease. Nat Rev Genet. 2008;9: 764–775. 10.1038/nrg2430 18802415 PMC3758473

