# Supplementary figures and images for: Widespread Changes in White Matter Microstructure after a Day of Waking and Sleep Deprivation
Source: PLoS One. 2015 May 28;10(5):e0127351. doi: 10.1371/journal.pone.0127351 (PMC4447359; doi:10.1371/journal.pone.0127351)

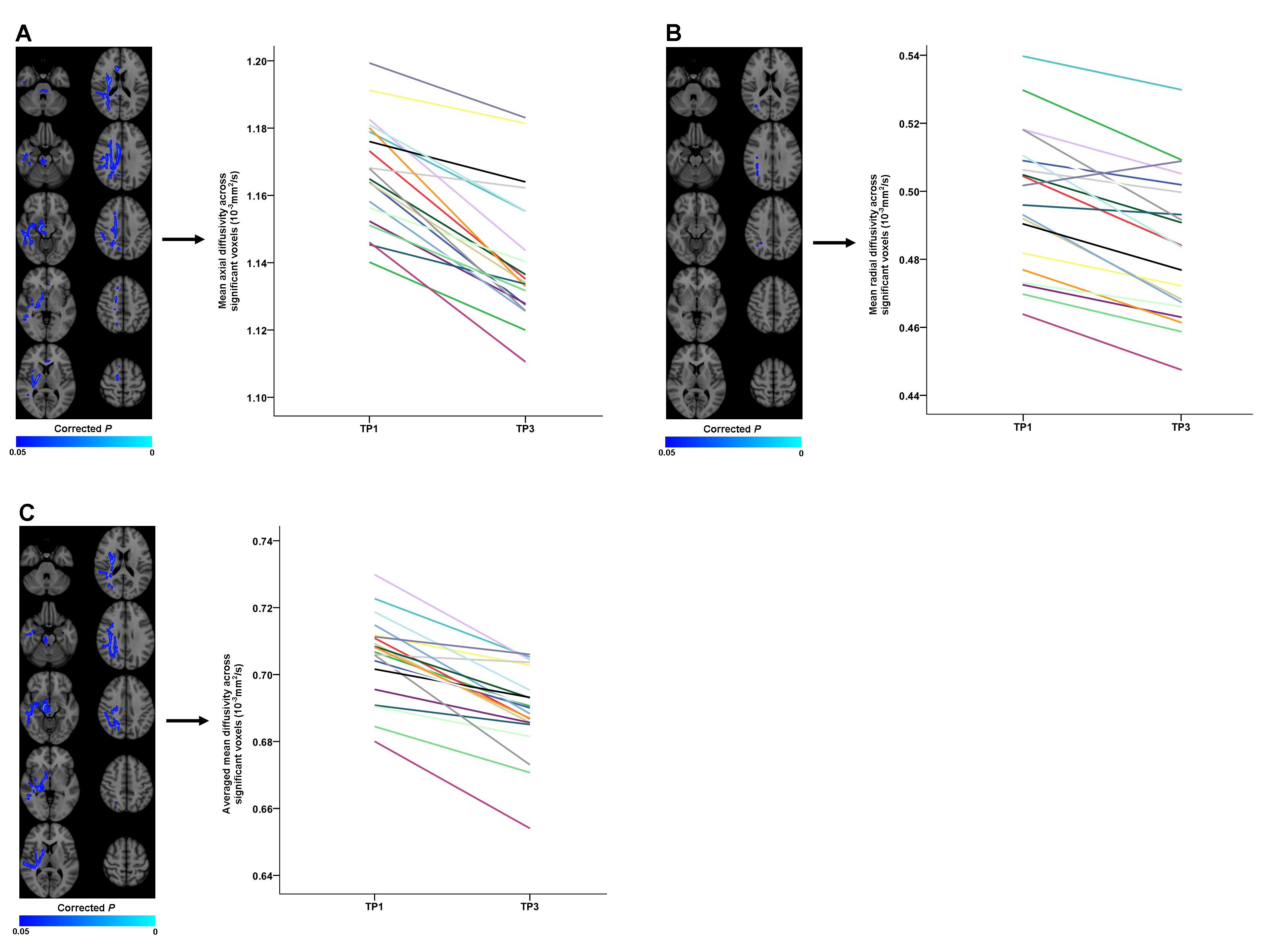

Supplement: S1 Fig — (A) Significant decreases in axial diffusivity after 23 hours of waking (blue colors; left panel). (B) Significant decreases in radial diffusivity after 23 hours of waking (blue colors; left panel). (C) Significant decreases in mean diffusivity after 23 hours of waking (blue colors; left panel). Averaged DTI values at time point (TP)1 and TP3 across significant voxels are shown for each participant using individual colors in the right panels of (A)–(C). Values from the same participant are connected with a line. The left side of the brain images represents the right hemisphere. (TIF) [file pone.0127351.s002.tif]
