# Supplementary material for: Widespread Changes in White Matter Microstructure after a Day of Waking and Sleep Deprivation
Source: PLoS One. 2015 May 28;10(5):e0127351. doi: 10.1371/journal.pone.0127351 (PMC4447359; doi:10.1371/journal.pone.0127351)
Supplement: S1 Table — (DOC) [file pone.0127351.s003.doc]

**S1 Table.** Clusters with significant changes in DTI indices of white matter microstructure after 23 hours of waking (TP1 compared with TP3).

| **DTI parameter** | **No. of voxels in cluster** | **Change after waking** | **MNI (x, y, z) maxima** | **Anatomical region of the peak voxel^a^** | **Peak voxel**  ***P*-value** |
| --- | --- | --- | --- | --- | --- |
| **AD** | 7704 | **↓** | 22, –6, 16 | R PLIC | 0.009 |
|  | 224 | **↓** | 7, –61, 26 | R Cingulum | 0.047 |
|  | 185 | **↓** | 34, –53, –11 | R ILF | 0.043 |
|  | 69 | **↓** | 37, –32, –23 | R Inferior temporal, fusiform* | 0.049 |
|  | 44 | **↓** | 43, –22, –3 | R ILF | 0.049 |
|  | 18 | **↓** | 5, 24, 13 | Fmaj | 0.049 |
| **RD** | 193 | **↓** | 36, –41, 31 | R SLF, ILF | 0.043 |
|  | 123 | **↓** | 37, –60, 30 | R Angular gyrus* | 0.046 |
|  | 23 | **↓** | 36, –67, 20 | R ILF, IFOF, Fmin | 0.048 |
|  | 12 | **↓** | 38, –71, 21 | R ILF, IFOF, Fmin | 0.049 |
|  | 5 | **↓** | 37, –19, 27 | R SLF | 0.049 |
| **MD** | 5727 | **↓** | 37, –36, 30 | R SLF | 0.027 |
|  | 633 | **↓** | 27, –40, 24 | R IFOF, ATR | 0.042 |
|  | 69 | **↓** | 29, –66, 23 | R IFOF, SLF, Fmaj | 0.048 |
|  | 6 | **↓** | 4, –7, 1 | R ATR | 0.049 |
|  | 5 | **↓** | 36, –48, 13 | R SLF, IFOF, ILF | 0.049 |

DTI; diffusion tensor imaging. TP; time point. MNI; Montreal Neurological Institute. R; right. L; left. PLIC; posterior limb of internal capsule. ILF; inferior longitudinal fasciculus. Fmaj; forceps major. SLF; superior longitudinal fasciculus. IFOF; inferior fronto-occipital fasciculus. Fmin; forceps minor. ATR; anterior thalamic radiation.

^a^Anatomical region based on Johns Hopkins University (JHU) white matter tractography atlas and the ICBM-DTI-81 white matter labels atlas .

*Not within the white matter atlases; gross anatomical description.

**Supporting references**

1. Hua K, Zhang J, Wakana S, Jiang H, Li X, Reich DS, et al. Tract probability maps in stereotaxic spaces: analyses of white matter anatomy and tract-specific quantification. Neuroimage. 2008;39: 336-347.

2. Wakana S, Jiang H, Nagae-Poetscher LM, van Zijl PC, Mori S. Fiber tract-based atlas of human white matter anatomy. Radiology. 2004;230: 77-87.

3. Mori S, Wakana S, Van Zijl PC, Nagae-Poetscher LM. MRI Atlas of Human White Matter. Amsterdam: Elsevier; 2005.
